# B cell depletion by Rituximab severely reduces immunoglobulin levels in patients with ANCA-associated vasculitis previously treated with cyclophosphamide

**DOI:** 10.1186/1479-5876-9-S2-P36

**Published:** 2011-11-23

**Authors:** Jens Thiel, Nora M Effelsberg, Klaus Warnatz, Michael Schlesier, Hans Hartmut Peter, Reinhard E Voll, Nils Venhoff

**Affiliations:** 1 Dept. of Rheumatology and Clinical Immunology, University Hospital Freiburg, Germany; 2 Centre for Chronic Immunodeficiency, University Hospital Freiburg, Germany

## Introduction

In ANCA-associated vasculitides (AAVs) cyclophosphamide (CYC) is part of the standard therapy regimen for severe manifestations. Rituximab (RTX) is effective in the treatment of AAVs. No data on the cumulative effect of a sequential treatment with CYC and rituximab on immunoglobulin levels in AAV are available. Such data are of great interest since both therapies can induce hypogammaglobulinemia leading to an increased risk of infections worsening the overall outcome.

## Objective and methods

Retrospective analysis of the impact of immunosuppressive therapy with CYC and RTX on serum immunoglobulin (Ig) concentrations and peripheral B cells in patients with AAV.

## Methods and patients

Ig levels were analyzed in AAV patients either treated with CYC alone or treated with CYC and RTX. The effect of CYC on Ig levels was assessed in study group A (n=26), while the impact of RTX treatment alone was examined in study group B (n=29). In patients previously treated with CYC followed by RTX we compared changes in Ig concentrations prior to CYC to those after RTX treatment (group C; n=15). The median CYC doses were 8.05±0.56 g, 18.76±14.55 and 13.11±13.27 g in groups A, B and C and median RTX doses in group B and C were 1.8±0.8 g and 1.97±1.04 g respectively. The mean prednisone equivalent in patients treated with RTX was 12.35±7.52 mg/day. Statistics: Mann Whitney U Test, if not indicated otherwise.

## Results

Mean Ig levels (mg/dL) prior to CYC therapy were normal. Means and standard deviations (SD) for group A are: IgG: 11.84±3.24, IgM: 1.31±0.94, IgA: 2.46±1.19. After CYC treatment IgG, IgM and IgA significantly decreased to 8.6±2.51 (p=<0.001; t-test), 0.88±0.53 (p=0.048), and 1.74±0.6 (p=0.021) respectively. In group B, median IgG levels prior to RTX were 9.74±3.49, IgM levels 0.81±0.45 and IgA levels 2.3±1.15. Over a period of 6 months after treatment, IgG significantly declined to 7.45±2.79 (p=0.001), IgM to 0.52±0.35 (p=0.003) and IgA to 1.69±0.81 (p=0.003). When assessing the effect of RTX therapy following CYC (group C) we observed the strongest decrease of all 3 isotypes: median IgG levels of 12.68±4.29mg/dL prior to CYC decreased to 6.59±1.98mg/dL (p=<0.001) after the dual treatment, IgM of 1.15±0.68 mg/dL to 0.52±0.42 mg/dL (p=0.002), and IgA of 2.65±1.25 mg/dL to 1.56±0.78 mg/dL (p=0.007). Follow-up measurements after RTX treatment did not show an increase in Ig levels within 24 months. RTX led to a complete depletion of B cells (</=2/µl) in all patients within the first 3 months after therapy. Interestingly, in all 7 patients that were available for analysis of peripheral B cell numbers 24 months after last RTX treatment, a persistent B lymphocytopenia with mean a B-cell count of 3.2/µl±1,7 was detected.

## Conclusions

In patients with AAV, treatment with CYC leads to a decline in immunoglobulin levels, which is profoundly aggravated by subsequent RTX treatment. Data in our cohort furthermore indicates that B cell repopulation in AAV patients after RTX treatment might be delayed. Hence, Ig concentrations should be assessed after combined treatment with CYC and RTX.

